# Supplementary material for: Patterns of Intron Gain and Loss in Fungi
Source: PLoS Biol. 2004 Nov 30;2(12):e422. doi: 10.1371/journal.pbio.0020422 (PMC532390; doi:10.1371/journal.pbio.0020422)
Supplement: Table S1 — Also available at http://genes.mit.edu/NielsenEtAl/. (4.3 MB ZIP). [file pbio.0020422.st001.zip › NielsenEtAl/html/472.html]

AN7770.1.NCU07958.1.MG05138.1.FG00407.1


```
 CLUSTAL W (1.82) Multiple Sequence Alignments - Introns Inserted


Sequence 1: NCU07958.1	317 aa
Sequence 2: MG05138.1	313 aa
Sequence 3: FG00407.1	307 aa
Sequence 4: AN7770.1	315 aa
Alignment Length: 326 aa
Number Identitical Residues: 143 aa
Alignment Score (without introns) 7295


MG05138.1 	MAT---EYMSGVWKDGIFK1DRVIFVTGGAGDICSAQTKAIVHLGANACIIGRNVEKTER
NCU07958.1	MSVPKSEYLSSVWAPGIFA1NRVVFVTGGAGTICSAQTRAMVHLGADACIIGRNPEKTEK
FG00407.1 	MSVPESEYLSPVWKDGIFN1GRVVFVTGGAGSICSMQTRALVRLGANACIVGRSVDKTEK
AN7770.1  	MPLPKSDYVSDSWKDGLFT1NKVVFCTGGAGTICSAQVRALVHLGADACIVGRNVEKTEK
          	*. ..::*:*  *  *:*  .:*:* ***** *** *.:*:*:***:***:**. :***:

MG05138.1 	VAREIAQVRPGAKVIGIGAVDVRK0FEDLKKAADRCASELGAIDFVI~AGAAGNFVAPIS
NCU07958.1	AAKDIAKVRSGARVIGIGNVDVRN0FDNLKAAAERCVKELGAIDFVI~AGAAGNFIAPIS
FG00407.1 	AAKEIESVREGAKVIAIGGCDVRK0VDSLQAAAERCAKELGGIDFVI2AGAAGNFVAPIE
AN7770.1  	MAQDIATARPGAKVVGIGAVDVRS~FGSLQSAVDRCVKEFGAIDYVI2AGAAGNFLASIN
          	 *::*  .* **:*:.**  ***. . .*: *.:**..*:*.**:** *******:*.*.

MG05138.1 	ALTPNGFKSVIDIDTIGTFNTIKATMPHLVASASR-----NPN~PSPTGQTGGRIIYVSA
NCU07958.1	GLSPNAFKTVIDIDTIGTFNTVKATIPYLIESAAR-----NPN~PNPNGLTGGRIISVSA
FG00407.1 	GLSSNAFKSVMDIDVLGTFNTVKATMPYLLRSS-------NP-~---------RIIYVSA
AN7770.1  	QISVNAFRSVMEIDVLGSYNTLKATIPHLVESAKKHRVDSNSL1KPSPAGTGGRIIFVSA
          	 :: *.*::*::**.:*::**:***:*:*: *:     .:*.    . . :..*** ***

MG05138.1 	TFHYTGVPLQAHVSAAKAGVDSLMASVAIEYGPLGITSNVISPGGIEGTE~GMERLSSGE
NCU07958.1	TFHYSGMPLQAHVSAAKAAVDSLMASVSLEYGPYGVTANVIAPGAIEGTE~GMERLASSA
FG00407.1 	TFHYTGMPLQAHVSAAKASVDSLMASVALEYGPRGVQSNVIAPGGIEGTE~GLARLGSDA
AN7770.1  	TLHYTGTPFQAHVAVAKAGIDALSHTVALEFGPLGVTSNVIAPGPIASTE0GLDRLLPSD
          	*:**:* *:****:.***.:*:*  :*::*:** *: :***:** * .** *: ** .. 

MG05138.1 	VNKDPKLGAAGVPTGRWGTKRDIGDATVFLFSEAGNYVNGHCLVV~DGAAWRGTNAG-VG
NCU07958.1	VDK--KKMTKAVPSGRWGSRRDIADATVYLFSDAGNYVNGTTLVV~DGAGWRRQGGNDIG
FG00407.1 	ESEK-KRYAKSIPLGRAGTVRDIADATVFLFSDAGSYVSGQVLAV~DGAAWRRQGALGVG
AN7770.1  	QLEK---AQRAQPLGRFGSVRDIADATVYLFADTGSYVSGQILVV1DGASWRISSAG-IG
          	  :.      . * ** *: ***.****:**:::*.**.*  *.* ***.**  ..  :*

MG05138.1 	LDPGMRYPDFIISGEFSKNIKSGRK--SKL--
NCU07958.1	IDEGMEYPDFLFTGQISKNLKDGRKQKSKL--
FG00407.1 	TEAGMEYPDYLLKGEFSQNLRDPRK-TAKVKL
AN7770.1  	SSN---YPDFLLSGDTFQNVKGKKK--AKL--
          	 .    ***:::.*:  :*::. :*  :*:
```
